# Antipsychotic Prescribing for In-Patients With Dementia at University Hospital Llandough to Look for Good Prescribing Practice in Line With NICE Guidelines

**DOI:** 10.1192/bjo.2024.619

**Published:** 2024-08-01

**Authors:** Rakesh Puli, Arpita Chakrabarti

**Affiliations:** Cardiff and Vale University Health Board, Cardiff, United Kingdom

## Abstract

**Aims:**

NICE guidelines stipulate that alternative causative factors for Behavioural and Psychiatric Symptoms of Dementia (BPSD) must be considered before starting antipsychotic treatment. The symptoms of BPSD include agitation, aggression, wandering, hoarding, sexual disinhibition, shouting, repeated questioning, sleep disturbance, depression, anxiety and psychosis. Those who do develop non-cognitive symptoms or behaviours should at first be assessed to exclude alternative causes, such as physical health issues (pain/infection), side effects of medication, environmental factors, psychosocial factors, individual biography (e.g. religious beliefs) etc. Then, non-pharmacological approaches should always be used as the first line in treating behavioural problems before antipsychotics (e.g. haloperidol or risperidone) are started at a low dose and titrated up. Once these have been started, the patient should be reviewed at 6 weeks. The rationale for conducting this audit is to try and understand if the antipsychotic prescribing in the ward is in line with the NICE guidelines.

**Methods:**

A retrospective study to compare the treatment of all the patients admitted for dementia in the Old age psychiatry wards located in University Hospital Llandough from November 2022–April 2023 with the NICE guidelines.

**Results:**

Out of the 39 patients who met the criteria, the results indicate a predominant prevalence of Alzheimer's (46%), followed by mixed dementia (23%) and vascular dementia (21%), among the diagnosed cases. In 67% of instances, healthcare professionals have considered alternative causative factors for the observed symptoms beyond the identified dementia subtypes. In 62% of cases, patients received treatment for alternative causes, while non-pharmacological approaches were attempted in 51%. The utilization rates among patients indicate a predominant prescription of risperidone at 77%, followed by quetiapine at 31%, olanzapine at 10%, and aripiprazole at 5%. 95% of patients were commenced treatment at the lowest dose, while information for 3% (1 patient) was not available. 62% were monitored according to guidelines and 56% were reviewed every 6 weeks.

**Conclusion:**

There is room for improvement in terms of considering other causes of behavioural symptoms, utilizing non-pharmacological approaches, and adherence to monitoring and review intervals outlined in the guidelines. These findings underscore the importance of continuous evaluation and refinement of clinical practices to enhance the overall management of BPSD in dementia patients.

